# DEXA Scan Body Fat Mass Distribution in Obese and Non-Obese Individuals and Risk of NAFLD—Analysis of 10,865 Individuals

**DOI:** 10.3390/jcm11206205

**Published:** 2022-10-21

**Authors:** Caitlyn Tan, Kai En Chan, Cheng Han Ng, Michael Tseng, Nicholas Syn, Ansel Shao Pin Tang, Yip Han Chin, Wen Hui Lim, Darren Jun Hao Tan, Nicholas Chew, Elden Yen Hng Ong, Teng Kiat Koh, Jieling Xiao, Douglas Chee, Arun Valsan, Mohammad Shadab Siddiqui, Daniel Huang, Mazen Noureddin, Karn Wijarnpreecha, Mark D. Muthiah

**Affiliations:** 1 Yong Loo Lin School of Medicine, National University of Singapore, Singapore 117597, Singapore; 2 Division of Gastroenterology, Hepatology and Nutrition, Department of Internal Medicine, Virginia Commonwealth University, Richmond, VA 23284, USA; 3 Department of Hematology-Oncology, National University Cancer Institute, Singapore 119074, Singapore; 4 Department of Cardiology, National University Heart Centre, National University Hospital, Singapore 119074, Singapore; 5 Division of Gastroenterology and Hepatology, Department of Medicine, National University Hospital, Singapore 119074, Singapore; 6 Department of Gastroenterology and Hepatology, Amrita Hospital, Kochi 682041, India; 7 National University Centre for Organ Transplantation, National University Health System, Singapore 119228, Singapore; 8 Houston Research Institute, Houston, TX 77079, USA; 9 Division of Gastroenterology and Hepatology, University of Arizona College of Medicine Phoenix, Phoenix, AZ 85004, USA

**Keywords:** non-alcoholic fatty liver disease, non-obese NAFLD, lean NAFLD, dual-energy X-ray absorptiometry, obesity

## Abstract

Non-alcoholic fatty liver disease (NAFLD) is the most common chronic liver disease worldwide yet predicting non-obese NAFLD is challenging. Thus, this study investigates the potential of regional fat percentages obtained by dual-energy X-ray absorptiometry (DXA) in accurately assessing NAFLD risk. Using the United States National Health and Nutrition Examination Survey (NHANES) 2011–2018, multivariate logistic regression and marginal analysis were conducted according to quartiles of regional fat percentages, stratified by gender. A total of 23,752 individuals were analysed. Males generally showed a larger increase in marginal probabilities of NAFLD development than females, except in head fat, which had the highest predictive probabilities of non-obese NAFLD in females (13.81%, 95%CI: 10.82–16.79) but the lowest in males (21.89%, 95%CI: 20.12–23.60). Increased percent of trunk fat was the strongest predictor of both non-obese (OR: 46.61, 95%CI: 33.55–64.76, *p* < 0.001) and obese NAFLD (OR: 2.93, 95%CI: 2.07–4.15, *p* < 0.001), whereas raised percent gynoid and leg fat were the weakest predictors. Ectopic fat deposits are increased in patients with non-obese NAFLD, with greater increases in truncal fat over gynoid fat. As increased fat deposits in all body regions can increase odds of NAFLD, therapeutic intervention to decrease ectopic fat, particularly truncal fat, may decrease NAFLD risk.

## 1. Introduction

Non-alcoholic fatty liver disease (NAFLD) is the most common chronic liver disease worldwide, affecting an estimated 33% of the global population [1,2,3]. NAFLD ranges in severity from a more benign hepatocyte fat accumulation in non-alcoholic fatty liver (NAFLD) without significant necroinflammation to non-alcoholic steatohepatitis (NASH) [4] where lobular inflammation, ballooning with or without fibrosis, can occur [5]. The pathogenesis of NAFLD results primarily from obesity and insulin resistance in the absence of substantial alcohol use [5] and is representative of systemic metabolic dysfunction that accompanies conditions such as type 2 diabetes mellitus, cardiovascular disease, and dyslipidaemia [6,7]. Obesity has classically been the principal risk factor for development of NAFLD [8]. Traditionally, it has been defined as a body mass index (BMI) ≥ 27.5 kg/m^2^ in Asians and BMI ≥ 30 kg/m^2^ in non-Asians [9], and the popularity of BMI in epidemiological studies likely stems from the ease of measuring height and weight; in excess of 50% of overweight and obese individuals are estimated to have NAFLD [10].

Studies have since revealed a significant proportion of the NAFLD population to be non-obese [11,12]. A recent prevalence meta-analysis indicated that 19.2% and 40.8% of NAFLD patients were lean and non-obese, respectively [13], and an estimated one in six NAFLD patients have a normal BMI [14]. Given that visceral fat mass has repeatedly been associated with NAFLD independent of BMI, this calls into question the one-size-fits-all model of using BMI to accurately assess obesity. Other more specific anthropometric indicators of ectopic fat have gained recognition as better predictors of NAFLD, with central obesity being more accurate than simple obesity [15] and dual-energy X-ray absorptiometry (DXA) a better adiposity measure than BMI [7]. Body fat composition and distribution may be a potential tool to assess the development of NAFLD [16] as compared with obesity alone, where the relationship between fat deposition patterns and NAFLD risk requires further clarification. Therefore, this study aims to investigate the predictive power of regional fat percentages on NAFLD risk in the context of obese as well as non-obese individuals, potentially improving risk stratification and prognostication in the non-obese population.

## 2. Materials and Methods

This study analyses patients recruited between 2011 and 2018 from NHANES, a health database consisting of responses to a clustered sampled national health survey. The full methodology of the NHANES study has been described elsewhere [17]. Participants of this cross-sectional survey platform underwent comprehensive interviews, medical examination, and laboratory assessments, and were representative of the general and non-institutionalized individuals in the United States between 2011 and 2018. As this present study uses de-identified data that have been publicly published by the National Centre for Health Statistics (NCHS), ethics approval by the Institutional Review Board was not required. Baseline characteristics such as age, gender, ethnicity, low-density lipoprotein (LDL) cholesterol, high-density lipoprotein (HDL) cholesterol, total cholesterol, triglyceride, total bilirubin, fasting blood glucose, glycohemoglobin, aspartate aminotransferase (AST), alanine aminotransferase (ALT), and past medical history (diabetes, hypertension, and obesity), were collected [18]. From the resulting participants, information on total, subtotal, android, head, trunk, gynoid, average arm, and average leg fat percentages based on the DEXA scans were collected.

The definition of NAFLD was adapted based on the American Association for the Study of Liver Disease (AASLD) guidelines for NAFLD [19]. We defined NAFLD as the presence of steatosis in the absence of substantial alcohol use (≥2 drinks a day in men, ≥3 drinks a day in women). The presence of steatosis in NAFLD was quantified with either fatty liver index (FLI) or United States Fatty Liver Index (US-FLI) with a cut-off of ≥60 [20] and ≥30 [21], respectively. Diabetes was defined as glycohemoglobin ≥ 6.5%, fasting plasma glucose ≥ 7mmol/L, self-reported diabetes, or the use of antidiabetic medications [22]. Non-invasive tests (NITs) for fibrosis include Aspartate Aminotransferase to Platelet Ratio Index (APRI), Fibrosis-4 (FIB-4) Index, and NAFLD Fibrosis Score. Obese patients were defined as BMI ≥ 27.5 kg/m^2^ for Asians and BMI ≥ 30 kg/m^2^ for other races [9]. Hypertension was defined as a systolic or diastolic blood pressure ≥ 130/85 or the use of antihypertensive medications [23]. 

Total and regional fat mass were measured by the whole-body scans acquired on the Hologic Discovery model A densitometers (Hologic, Inc., Bedford, MA, USA) and analysed using software version Apex 3.2. The DXA examinations were administered by trained and certified radiology technologists who were blinded to the patient characteristics and laboratory data. DXA scanning was applied in a supine position with no movement according to the manufacturer’s instructions. A whole-body DXA examination included total body and regional measurements of the right and left arms, left and right legs, head, trunk, and abdomen. All fat measurements were expressed as a percentage of the total body mass. The android fat region was defined by the Hologic APEX software as the lower trunk area bounded by two lines: the pelvic horizontal cut line on its lower side, and a line automatically placed above the pelvic line. The gynoid region was demarcated by a higher gynoid line placed 1.5 times of the height of android region below the pelvic line, and the lower gynoid line was placed such that the distance between the two gynoid lines was twice the height of the android region. The above-mentioned lines were automatically placed by Hologic software [24].

All statistical analysis was conducted in STATA (16.1). A *p*-value ≤ 0.05 was considered as the threshold for statistical significance. Descriptive statistics were summarized in median and interquartile range (IQR) for continuous variables and proportions with 95% confidence intervals for binary variables. Continuous variables were examined with the ranked sum test while binary variables were examined with chi-square test and Fisher exact test, where appropriate. Kendall correlation coefficients between baseline regional fat measures in males and females were calculated. A multivariate logistic regression model was estimated to determine independent associations between regional body fat percentages and risk of developing NAFLD according to quartiles of the respective body fat measure. A cluster analysis was included based on the year of study to account for heterogeneity introduced by the year of study. Multivariable model in logistic regression was constructed with important traditional confounders including age, gender, diabetic status, and ethnicity, and a marginal model analysis was conducted to estimate the predictive probability of regional body fat percentages and risk of NAFLD development, stratified by gender. Logistic regression and marginal analysis were conducted according to quartiles of regional body fat percentages.

## 3. Results

### 3.1. Baseline Characteristics of Non-Obese NAFLD vs. Non-NAFLD 

In total, 23,752 patients were included in the analysis. The baseline demographics of the non-obese population is summarized in Table 1. Among these participants, 1347 (11.40%) had NAFLD, while 10,474 (88.60%) did not, where older age and male gender appear to be predisposing factors of NAFLD. Additionally, NAFLD patients were significantly more likely to exhibit conditions commonly associated with systemic metabolic dysfunction compared with their non-NAFLD counterparts. Notably, the prevalence of diabetes in NAFLD patients was 23.40% (95%CI: 21.18–25.78) compared with 9.91% (95%CI: 9.34–10.50) in non-NAFLD. NAFLD (60.06%, 95%CI: 57.36–62.71) patients were also found to be significantly more hypertensive than non-NAFLD (37.20%, 95%CI: 36.25–38.16) individuals. Not surprisingly, the lipid profiles of individuals with NAFLD were significantly worse than non-NAFLD patients (*p* < 0.01). Regional body fat distribution measurements were found to be significantly higher in NAFLD than non-NAFLD patients, except those of gynoid fat which was not associated with a statistical difference (*p* = 0.13).

### 3.2. Risk of NAFLD in Non-Obese Individuals

**Figure 1:**
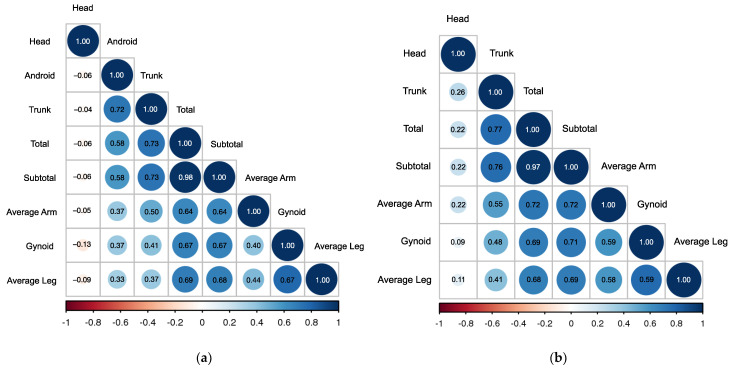
Correlation matrix of Fat Distribution in Non-obese (**a**) Female NAFLD (**b**) Male NAFLD.

A correlation matrix of the fat distribution in non-obese NAFLD individuals is summarized in Figure 1a,b for female and male gender, respectively. As seen in Table 2, there was a lack of strong correlation across all measures of body fat, except between total and subtotal fat percentages in males (r = 0.97) and females (r = 0.98). All body fat percentages in both genders were positively correlated with one another, except head fat in females, which was inversely correlated with all other body regions. 

**Figure 2:**
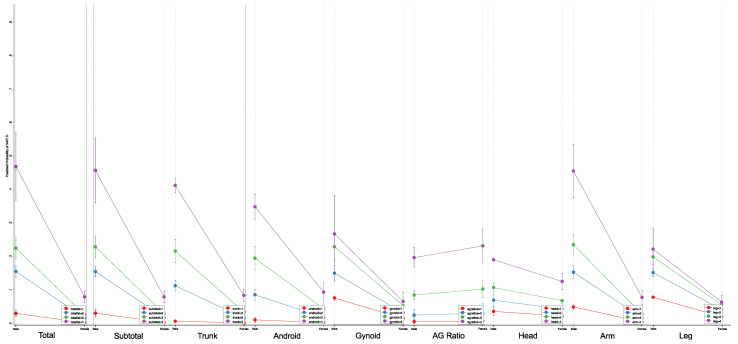
Predictive Probability of Regional Body Fat Percentages and Risk of NAFLD Development in Non-obese Population.

**Figure 3:**
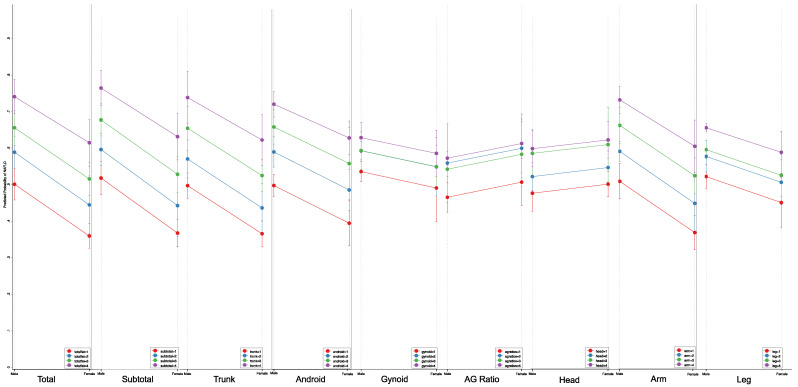
Predictive Probability of Regional Body Fat Percentages and Risk of NAFLD Development in Obese Population.

A multivariate logistic regression analysis adjusted for age, race, BMI, and diabetic status clustered on the study year was used to examine the risk of NAFLD and body fat distribution in non-obese (Figure 2) and obese (Figure 3) populations. In both populations, all quartiles of body fat percentages were significantly (*p* < 0.001) and positively associated with an increased risk of NAFLD. With reference to Q1 of body fat distribution, NAFLD risk increased with higher body fat percentages from Q2 to Q4, where each standard deviation increment in all regional measurements was positively associated with NAFLD risk. In the non-obese population, Q4 of total, subtotal, trunk, android, and average arm fat percentages were associated with significant odds of NAFLD development compared with Q1. Greater percentages of leg (OR: 3.19, 95%CI: 1.85–5.49, *p* < 0.001) and gynoid (OR: 3.83, 95%CI: 1.90–7.73, *p* < 0.001) fat showed the smallest magnitudes of effect in non-obese individuals, albeit being significantly associated with increased risk of NAFLD. 

Thereafter, a marginal model was constructed to examine the influence of fat distribution by quartiles and the predicted probability of NAFLD, segregated by gender in the non-obese (Table 3) population. In non-obese individuals, males were shown to have a higher probability of NAFLD than females in each quartile of body fat percentage. Furthermore, the association between increasing fat mass and NAFLD risk was considerably more prominent in males than females. Percent leg fat was associated with one of the lowest marginal probabilities (22.25%, 95%CI: 15.75–28.74) of NAFLD in Q4 of percent body fat. Of all regional body fat measures in males, Q4 of truncal fat was associated with the highest predictive probability of NAFLD (56.57%, 95%CI: 53.37–59.75), four times the corresponding probability of NAFLD associated with trunk fat in females (13.21%, 95%CI: 10.11–16.30). Interestingly, Q4 of head fat demonstrated the highest predictive probability of NAFLD in females (13.81%, 95%CI: 10.82–16.79), while simultaneously having the lowest associated marginal probability in males (21.89%, 95%CI: 20.12–23.60).

### 3.3. Risk of NALFD in Obese Individuals

**Figure 4:**
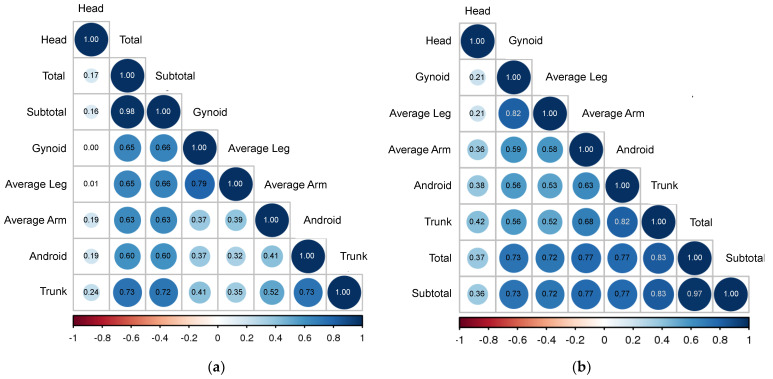
Correlation matrix of Fat Distribution in Obese (**a**) Female NAFLD (**b**) Male NAFLD.

A correlation matrix of the fat distribution in obese NAFLD individuals is summarized in Figure 4a,b for female and male gender, respectively. From Table 4, leg fat was positively correlated with gynoid fat in both genders. Males showed stronger overall correlations between body fat regions than females, whereas trunk fat in males was significantly and positively correlated with total (r = 0.83), subtotal (r = 0.83) and android (r = 0.82) fat regions. Trunk fat in females was not correlated with any other body fat region. 

As can be seen from Table 5, the multivariate logistic regression model presented a consistent trend of rising body fat percentages being associated with increased odds of NAFLD development in obese individuals. The Q4 of percent trunk fat was associated with the highest odds of NAFLD development for both non-obese (OR: 46.61, 95%CI: 33.55–64.76, *p* < 0.001) and obese individuals (OR: 2.93, 95%CI: 2.07–4.15, *p* < 0.001) relative to their respective Q1 of body fat distribution. From the marginal model (Table 5), obese males also generally displayed a higher probability of NAFLD than obese females. The only exception was percent head fat, where it demonstrated a consistently higher predictive probability of developing NAFLD in females than males across all body fat quartiles. Among all body regions, Q1 of percent head fat was most strongly associated with NAFLD risk in obese females (49.46%, 95%CI: 46.05–52.86), while being associated with the lowest probability of NAFLD development in obese males (47.00%, 95%CI: 32.14–51.87).

Similar to the non-obese population, percent gynoid and leg fat in Q4 of body fat distribution were most weakly associated with NAFLD development in the obese population, whereas total and trunk fat percentages demonstrated the strongest predictive margins of NAFLD development. With greater accumulation of fat in all regional fat percentages in Q4 of obese individuals, however, there was a relatively smaller distinction in the predictive probability of NAFLD between different body fat regions than in non-obese individuals. In obese females, gynoid fat in Q4 was most weakly associated with NAFLD risk at 58.13% (95%CI: 51.83–64.43), compared to percent trunk fat which demonstrated the highest predictive probability of NAFLD development at 62.45% (95%CI: 55.51–69.39). In contrast, in non-obese females, the predictive margins of NAFLD developments were weakest in percent gynoid fat (6.34%, 95%CI: 3.75–8.91) and highest in head fat (13.81%, 95%CI: 10.82–16.79). 

## 4. Discussion

The challenges of managing NAFLD are heightened in patients with non-obese NAFLD. In this study, we correlated ectopic adipose tissue deposits to NAFLD in non-obese patients to shed light on the role of these deposits in the development of non-obese NAFLD. The key finding from our study was that in patients with non-obese NAFLD, these ectopic fat deposits were found to be higher. Of note, all the regional ectopic fat deposits were increased in patients with non-obese NAFLD, except for gynoid fat. This is in keeping with the current literature in NAFLD, where patients with NAFLD were associated with an increase in the android-fat-to-gynoid-fat ratio (AGR) [25]. The android fat deposits are in the midsection of the abdomen and contribute to the metabolically active visceral fat [26]. Gynoid fat, on the other hand, is found around the hips and has been associated with an improved cardiometabolic profile, as well as being protective against NAFLD [27]. Furthermore, Ciardullo et al. [28] recently found that AGR was significantly associated with liver fibrosis as determined by transient elastography in females, whereas this association was statistically insignificant in males. While the pathophysiology underlying this gender discrepancy requires further investigation, this finding points toward the predictive potential of AGR beyond NAFLD development to act as a surrogate marker for NAFLD-related significant fibrosis.

Interestingly, the association between head fat and non-obese NAFLD was discordant between males and females. Head fat had the highest predictive probability of non-obese NAFLD in females, while it had the lowest predictive probability in males. This aligns with previous studies where the only DXA measure that improved the prediction of insulin sensitivity was percent head fat, and only in females [29]. Additionally, head fat has been positively correlated with upper limb fat, trunk fat, fasting plasma insulin, and uric acid in females [30]. Despite its significant association with different anthropological and metabolic variables in women, the current implications of head fat remain to be ascertained and may provide an interesting angle to understanding disease in NAFLD. However, current available data on its association with NAFLD are limited, but this novel information does warrant further study. 

Importantly, the marginal effect of ectopic fat distributions on the predictive probability of having NAFLD were more significant in non-obese NAFLD relative to obese NAFLD. The importance of weight remains key in the management principle for NAFLD [31,32]. However, there should also be a wholesome reduction in fat mass beyond visceral fats which are most commonly associated with NAFLD. While the ectopic fat deposits have been associated in patients with NAFLD, this is the first study to investigate the ectopic fat deposits in patients with non-obese NAFLD. This demonstrates the role of ectopic and visceral fat in the development of non-obese NAFLD, placing importance on the truncal regions of adiposity despite these individuals being non-obese. It is challenging to set weight loss targets for patients with non-obese and lean NAFLD; in addition, the association of these lipid deposits with NAFLD does bring up the possibility of using some of these indices as targets for weight loss in such populations and may be enhanced by the availability of new technologies in body composition imaging [33].

## 5. Limitations

This study presents a comprehensive review of the associations between body fat distribution and NAFLD development through a population analysis of 10,865 individuals. However, there are several limitations to this study. Firstly, the NHANES dataset was used in a cross-sectional evaluation of NAFLD patients, thus making it impossible to assess temporal causality inference. Additionally, hepatic steatosis was defined by FLI and may be suboptimal as compared with biopsy and imaging-based diagnoses. There were also insufficient data to assess alcohol-binging history and confounding reasons which may contribute to hepatic fat accumulation. Nevertheless, the study still alludes to the value of ectopic fat deposits in the clinical management of NAFLD.

## 6. Conclusions

The current study examines the weight distribution in NAFLD and the implications on risk of fatty liver. Importantly, fat distribution in all corners of the body can result in increased odds of fatty liver. Ectopic fat deposits are increased in patients with non-obese NAFLD, with an increase in truncal fat over gynoid fat. Further study is warranted to determine their usefulness in the clinical management of NAFLD. Therapeutic intervention to decrease ectopic fat, particularly truncal fat, may decrease the risk of NAFLD.

## Figures and Tables

**Table 1:** Baseline Demographics of NAFLD and Non-NAFLD Patients in Non-obese Population.

	NAFLD	Non-NAFLD	*p*-Value
Sample Size	1347	10,474	<0.01 *
Age (years)	58.00 (IQR: 44.00 to 68.00)	44.00 (IQR: 29.00 to 62.00)	<0.01 *
Gender (male)	65.40 (95%CI: 62.82 to 67.90)	49.23 (95%CI: 48.28 to 50.19)	<0.01 *
Platelet (1000 cells/μL)	230.00 (IQR: 195.00 to 264.00)	228.00 (IQR: 195.00 to 265.00)	0.81
Glycohemoglobin (%)	5.70 (IQR: 5.40 to 6.10)	5.40 (IQR: 5.20 to 5.70)	<0.01 *
Fasting Glucose (mmol/L)	5.94 (IQR: 5.50 to 6.72)	5.44 (IQR: 5.05 to 5.88)	<0.01 *
Total Bilirubin (μmol/L)	10.26 (IQR: 6.84 to 13.68)	10.26 (IQR: 6.84 to 13.68)	0.92
AST (IU/L)	25.00 (IQR: 21.00 to 30.00)	21.00 (IQR: 18.00 to 25.00)	<0.01 *
ALT (IU/L)	28.00 (IQR: 21.00 to 38.00)	18.00 (IQR: 14.00 to 24.00)	<0.01 *
LDL (mg/dL)	120.00 (IQR: 94.50 to 146.00)	105.00 (IQR: 84.00 to 129.00)	<0.01 *
HDL (mg/dL)	45.00 (IQR: 38.00 to 53.00)	55.00 (IQR: 46.00 to 66.00)	<0.01 *
Total Cholesterol (mg/dL)	202.00 (IQR: 178.00 to 232.00)	182.00 (IQR: 157.00 to 210.00)	<0.01 *
Triglyceride (mg/dL)	208.00 (IQR: 143.00 to 294.00)	97.00 (IQR: 67.00 to 143.00)	<0.01 *
Diabetes	23.40 (95%CI: 21.18 to 25.78)	9.91 (95%CI: 9.34 to 10.50)	<0.01 *
Hypertension	60.06 (95%CI: 57.36 to 62.71)	37.20 (95%CI: 36.25 to 38.16)	<0.01 *
Total Fat (%)	29.90 (IQR: 26.80 to 36.55)	29.10 (IQR: 23.60 to 35.80)	<0.01 *
Subtotal Fat (%)	30.20 (IQR: 27.00 to 37.50)	29.60 (IQR: 23.60 to 36.80)	<0.01 *
Android Fat (%)	36.30 (IQR: 32.50 to 40.40)	30.90 (IQR: 24.40 to 36.70)	<0.01 *
Head Fat (%)	24.20 (IQR: 23.90 to 24.50)	23.80 (IQR: 23.50 to 24.10)	<0.01 *
Trunk Fat (%)	31.10 (IQR: 27.90 to 36.60)	27.40 (IQR: 21.70 to 33.50)	<0.01 *
Average Arm Fat (%)	27.55 (IQR: 24.05 to 38.25)	28.30 (IQR: 21.20 to 38.60)	<0.01 *
Average Leg Fat (%)	29.45 (IQR: 25.85 to 37.20)	32.55 (IQR: 24.60 to 40.75)	<0.01 *
Gynoid Fat (%)	30.90 (IQR: 27.70 to 37.60)	33.30 (IQR: 26.30 to 40.60)	0.13
Ethnicity			<0.01 *
Mexican American	20.71 (95%CI: 18.63 to 22.96)	11.86 (95%CI: 11.25 to 12.49)	
Hispanic	12.18 (95%CI: 10.53 to 14.03)	10.61 (95%CI: 10.03 to 11.21)	
White	42.61 (95%CI: 40.00 to 45.27)	37.77 (95%CI: 36.85 to 38.70)	
Black	13.07 (95%CI: 11.37 to 14.97)	19.58 (95%CI: 18.83 to 20.35)	
Other Race	11.43 (95%CI: 9.84 to 13.25)	20.18 (95%CI: 19.43 to 20.96)	

Legend: NAFLD, Non-alcoholic Fatty Liver Disease; AST, Aspartate Aminotransferase; ALT, Alanine Aminotransferase; IQR, Interquartile Range; 95%CI, 95% Confidence Interval; LDL, Low Density Lipoprotein; and HDL, High Density Lipoprotein; * bolded *p*-value ≤ 0.01 denotes statistical significance.

**Table 2:** Correlations between Regional Body Fat Measures in Non-obese Males and Females.

	% Total Fat	% Subtotal Fat	% Trunk Fat	% Android Fat	% Gynoid Fat	% Head Fat	% Average Arm Fat	% Average Leg Fat
	Females			Both Genders		Males		
% Total Fat	**1.00**	0.97	0.77	-	0.69	0.22	0.72	0.68
% Subtotal Fat	0.98	**1.00**	0.76	-	0.71	0.22	0.72	0.69
% Trunk Fat	0.73	0.73	**1.00**	-	0.48	0.26	0.55	0.41
% Android Fat	0.58	0.58	0.72	**1.00**	-	-	-	-
% Gynoid Fat	0.67	0.67	0.41	0.37	**1.00**	0.09	0.59	0.59
% Head Fat	−0.06	−0.06	−0.04	−0.06	−0.13	**1.00**	0.22	0.11
% Average Arm Fat	0.64	0.64	0.50	0.37	0.40	−0.05	**1.00**	0.58
% Average Leg Fat	0.69	0.68	0.37	0.33	0.78	−0.09	0.44	**1.00**

**Table 3:** Multivariate Analysis of Body Fat on Risk of NAFLD Development in Non-obese Population.

	Quartile for Body Fat					
	Q1	Q2	Q3	Q4	*p* for Trend	Each SD Increment
**% Total Fat**					<0.001 *	
OR (95% CI)	Reference	3.89 (3.18 to 4.75)	6.83 (5.89 to 7.93)	19.03 (13.78 to 26.27)		4.03 (3.51 to 4.60)
Male [Margin (95%CI)]	6.32 (5.67 to 6.97)	19.60 (16.87 to 22.33)	29.04 (26.45 to 31.63)	50.80 (45.38 to 56.21)		
Female [Margin (95%CI)]	0.65 (0.42 to 0.88)	2.46 (1.47 to 3.44)	4.20 (3.09 to 5.31)	10.54 (8.05 to 13.02)		
**% Subtotal Fat**					<0.001 *	
OR (95% CI)	Reference	3.84 (3.20 to 4.60)	6.14 (4.85 to 7.77)	16.99 (10.99 to 26.28)		4.03 (3.52 to 4.61)
Male [Margin (95%CI)]	6.41 (5.63 to 7.00)	19.65 (17.03 to 22.26)	27.37 (24.03 to 30.72)	48.62 (41.14 to 56.11)		
Female [Margin (95%CI)]	0.72 (0.43 to 0.99)	2.66 (1.54 to 3.77)	4.16 (2.93 to 5.38)	10.38 (7.88 to 12.87)		
**% Trunk Fat**					<0.001 *	
OR (95% CI)	Reference	7.47 (5.66 to 9.86)	13.72 (10.37 to 18.15)	46.61 (33.55 to 64.76)		4.89 (4.54 to 5.27)
Male [Margin (95%CI)]	3.10 (2.18 to 4.02)	18.63 (15.97 to 21.28)	29.05 (24.14 to 33.96)	56.57 (53.37 to 59.75)		
Female [Margin (95%CI)]	0.34 (0.17 to 0.50)	2.45 (1.74 to 3.16)	4.39 (2.53 to 6.25)	13.21 (10.11 to 16.30)		
**% Android Fat**					<0.001 *	
OR (95% CI)	Reference	7.16 (4.64 to 11.05)	15.96 (11.09 to 22.98)	31.12 (23.80 to 40.68)		3.96 (3.66 to 4.28)
Male [Margin (95%CI)]	2.80 (1.60 to 4.00)	16.51 (14.38 to 18.63)	29.81 (28.59 to 31.03)	44.35 (41.20 to 47.49)		
Female [Margin (95%CI)]	0.47 (0.17 to 0.76)	3.22 (2.50 to 3.93)	6.83 (4.95 to 8.70)	12.31 (8.34 to 16.28)		
**Android-to-Gynoid Fat Ratio**					<0.001 *	
OR (95% CI)	Reference	2.28 (2.06 to 2.53)	3.30 (2.75 to 3.95)	3.85 (2.59 to 5.73)		-
Male [Margin (95%CI)]	7.50 (7.35 to 7.65)	14.87 (13.67 to 16.07)	19.58 (17.22 to 21.95)	19.58 (15.69 to 28.09)		
Female [Margin (95%CI)]	1.71 (1.24 to 2.18)	3.76 (2.43 to 5.08)	5.26 (3.26 to 7.27)	6.06 (4.15 to 7.97)		
**% Gynoid Fat**					<0.001 *	
OR (95% CI)	Reference	2.41 (2.10 to 2.76)	3.27 (2.08 to 5.14)	3.83 (1.90 to 7.73)		2.16 (1.86 to 2.51)
Male [Margin (95%CI)]	8.81 (7.52 to 10.09)	17.93 (16.95 to 18.90)	22.33 (15.90 to 28.75)	24.89 (15.74 to 34.03)		
Female [Margin (95%CI)]	1.80 (1.13 to 2.46)	4.16 (3.08 to 5.23)	5.49 (2.89 to 8.09)	6.34 (3.75 to 8.91)		
**% Head Fat**					<0.001 *	
OR (95% CI)	Reference	2.37 (1.99 to 2.82)	4.09 (3.46 to 4.84)	6.26 (6.02 to 6.51)		1.93 (1.86 to 2.00)
Male [Margin (95%CI)]	4.64 (4.31 to 4.97)	10.07 (9.00 to 11.13)	15.86 (14.49 to 17.20)	21.89 (20.12 to 23.60)		
Female [Margin (95%CI)]	2.63 (2.03 to 3.24)	5.92 (4.39 to 7.44)	9.66 (8.50 to 10.80)	13.81 (10.82 to 16.79)		
**% Average Arm Fat**					<0.001 *	
OR (95% CI)	Reference	3.17 (2.56 to 3.93)	4.65 (3.20 to 6.75)	11.72 (6.12 to 22.45)		3.82 (3.51 to 4.16)
Male [Margin (95%CI)]	7.47 (7.03 to 7.90)	19.31 (16.73 to 21.88)	25.36 (19.09 to 31.62)	43.90 (30.14 to 57.65)		
Female [Margin (95%CI)]	0.92 (0.53 to 1.30)	2.82 (1.27 to 4.36)	4.05 (3.43 to 4.66)	9.34 (6.12 to 12.55)		
**% Average Leg Fat**					<0.001 *	
OR (95% CI)	Reference	2.19 (1.75 to 2.74)	2.58 (1.62 to 4.12)	3.19 (1.85 to 5.49)		1.86 (1.58 to 2.17)
Male [Margin (95%CI)]	9.00 (8.07 to 9.92)	16.93 (14.65 to 19.20)	19.15 (14.07 to 24.22)	22.25 (15.75 to 28.74)		
Female [Margin (95%CI)]	2.19 (1.30 to 3.07)	4.57 (2.63 to 6.51)	5.32 (3.91 to 6.73)	6.43 (4.30 to 8.55)		

Legend: NAFLD, Non-alcoholic Fatty Liver Disease; 95%CI, 95% Confidence Interval; and OR, Odds Ratio. * bolded *p*-value ≤ 0.001 denotes statistical significance.

**Table 4:** Correlations between Regional Body Fat Measures in Obese Males and Females.

	% Total Fat	% Subtotal Fat	% Trunk Fat	% Android Fat	% Gynoid Fat	% Head Fat	% Average Arm Fat	% Average Leg Fat
		Females		Both Genders			Males	
% Total Fat	**1.00**	0.97	0.83	0.77	0.73	0.37	0.77	0.72
% Subtotal Fat	0.98	**1.00**	0.83	0.77	0.73	0.36	0.77	0.72
% Trunk Fat	0.73	0.72	**1.00**	0.82	0.56	0.42	0.68	0.52
% Android Fat	0.60	0.60	0.73	**1.00**	0.56	0.38	0.63	0.53
% Gynoid Fat	0.65	0.66	0.41	0.37	**1.00**	0.21	0.59	0.82
% Head Fat	0.17	0.16	0.24	0.19	0.00	**1.00**	0.36	0.21
% Average Arm Fat	0.63	0.63	0.52	0.41	0.37	0.19	**1.00**	0.58
% Average Leg Fat	0.65	0.66	0.35	0.32	0.79	0.01	0.39	**1.00**

**Table 5:** Multivariate Analysis of Body Fat on Risk of NAFLD Development in Obese Population.

	Quartile for Body Fat					
	Q1	Q2	Q3	Q4	*p* for Trend	Each SD Increment
**% Total Fat**					<0.001 *	
OR (95% CI)	Reference	1.43 (1.20 to 1.72)	1.92 (1.72 to 2.14)	2.90 (2.50 to 3.36)		1.51 (1.49 to 1.54)
Male [Margin (95%CI)]	50.10 (54.53 to 63.31)	58.87 (54.43 to 63.31)	65.57 (60.80 to 70.35)	74.08 (69.40 to 78.76)		
Female [Margin (95%CI)]	35.93 (36.49 to 52.38)	44.44 (36.49 to 52.38)	51.55 (47.02 to 56.09)	61.47 (55.04 to 67.89)		
**% Subtotal Fat**					<0.001 *	
OR (95% CI)	Reference	1.37 (1.14 to 1.65)	1.93 (1.75 to 2.13)	2.93 (2.59 to 3.33)		1.51 (1.48 to 1.54)
Male [Margin (95%CI)]	50.61 (46.10 to 55.12)	58.25 (53.88 to 62.61)	66.17 (61.88 to 70.45)	74.71 (69.97 to 79.44)		
Female [Margin (95%CI)]	35.86 (32.21 to 39.51)	43.22 (34.87 to 51.57)	51.62 (46.84 to 56.39)	61.69 (55.48 to 67.89)		
**% Trunk Fat**					<0.001 *	
OR (95% CI)	Reference	1.35 (1.06 to 1.72)	1.94 (1.60 to 2.36)	2.93 (2.07 to 4.15)		1.49 (1.39 to 1.59)
Male [Margin (95%CI)]	49.89 (46.40 to 53.38)	57.23 (52.01 to 62.45)	65.69 (59.66 to 71.72)	74.13 (66.92 to 81.34)		
Female [Margin (95%CI)]	36.64 (33.15 to 40.14)	43.74 (37.10 to 50.39)	52.65 (48.31 to 57.00)	62.45 (55.51 to 69.39)		
**% Android Fat**					<0.001 *	
OR (95% CI)	Reference	1.45 (1.24 to 1.70)	1.95 (1.65 to 2.32)	2.62 (2.46 to 2.78)		1.41 (1.34 to 1.47)
Male [Margin (95%CI)]	49.45 (46.44 to 52.46)	58.58 (54.49 to 62.68)	65.39 (60.74 to 70.05)	71.60 (68.09 to 75.11)		
Female [Margin (95%CI)]	39.19 (33.09 to 45.29)	48.24 (42.60 to 53.88)	55.35 (45.62 to 65.28)	62.40 (57.80 to 67.01)		
**% Gynoid Fat**					<0.001 *	
OR (95% CI)	Reference	1.26 (1.14 to 1.41)	1.27 (1.11 to 1.45)	1.47 (1.32 to 1.64)		1.27 (1.25 to 1.29)
Male [Margin (95%CI)]	53.18 (50.46 to 55.89)	58.85 (56.33 to 61.37)	58.90 (53.83 to 63.96)	62.41 (58.27 to 66.55)		
Female [Margin (95%CI)]	48.71 (39.58 to 57.85)	54.47 (46.56 to 62.32)	54.51 (48.70 to 60.32)	58.13 (51.83 to 64.43)		
**Android-to-Gynoid Fat Ratio**					<0.001 *	
OR (95% CI)	Reference	1.25 (1.16 to 1.35)	1.35 (1.22 to 1.50)	1.76 (1.61 to 1.92)		-
Male [Margin (95%CI)]	51.92 (48.70 to 55.13)	57.41 (53.16 to 61.67)	59.26 (56.79 to 61.74)	65.23 (64.08 to 66.39)		
Female [Margin (95%CI)]	44.82 (38.12 to 51.52)	50.35 (44.27 to 56.43)	52.25 (46.56 to 57.94)	58.52 (52.76 to 64.29)		
**% Head Fat**					<0.001 *	
OR (95% CI)	Reference	1.20 (1.14 to 1.27)	1.56 (1.14 to 2.12)	1.64 (1.53 to 1.77)		1.21 (1.16 to 1.25)
Male [Margin (95%CI)]	47.00 (32.14 to 51.87)	51.51 (47.77 to 55.24)	57.80 (51.32 to 64.29)	59.08 (54.16 to 63.99)		
Female [Margin (95%CI)]	49.46 (46.05 to 52.86)	53.95 (49.48 to 58.42)	60.18 (50.08 to 70.28)	61.43 (56.51 to 66.36)		
**% Average Arm Fat**					<0.001 *	
OR (95% CI)	Reference	1.40 (1.17 to 1.68)	1.91 (1.78 to 2.05)	2.66 (2.23 to 3.18)		1.50 (1.41 to 1.59)
Male [Margin (95%CI)]	50.55 (45.76 to 55.35)	58.75 (56.02 to 61.47)	65.86 (60.90 to 70.83)	72.82 (69.20 to 76.43)		
Female [Margin (95%CI)]	36.55 (31.84 to 41.26)	44.52 (36.91 to 52.13)	52.08 (47.23 to 56.93)	60.13 (52.97 to 67.29)		
**% Average Leg Fat**					<0.001 *	
OR (95% CI)	Reference	1.25 (1.16 to 1.35)	1.35 (1.22 to 1.50)	1.76 (1.61 to 1.92)		1.30 (1.28 to 1.33)
Male [Margin (95%CI)]	51.92 (48.70 to 55.13)	57.41 (53.16 to 61.67)	59.26 (56.79 to 61.74)	65.23 (64.08 to 66.39)		
Female [Margin (95%CI)]	44.82 (38.12 to 51.52)	50.35 (44.27 to 56.43)	52.25 (46.56 to 57.94)	58.52 (52.76 to 64.87)		

Legend: NAFLD, Non-alcoholic Fatty Liver Disease; 95%CI, 95% Confidence Interval; and OR, Odds Ratio. * bolded *p*-value ≤ 0.001 denotes statistical significance.

## Data Availability

Data were retrieved from the National Health and Nutrition 276 Examination Survey Registry.
